# Letter Regarding Adade and Das “Investigation of Selective Innervation of Extraocular Muscle Compartments”

**DOI:** 10.1167/iovs.64.7.38

**Published:** 2023-06-28

**Authors:** Joseph L. Demer, Robert A. Clark

**Affiliations:** 1 UCLA, Los Angeles, California, United States. E-mail: jld@jsei.ucla.edu.

This letter responds to the recent paper by Adade and Das, whose running head is titled “Testing the Theory of EOM Compartmentalization.”^1^ We are pleased that Adade and Das accepted the challenge in our 2016 paper in *Journal of Neurophysiology* to use single unit neural recordings to investigate selective compartmental function in the extraocular muscles (EOMs) of non-human primates.^2^ These investigators are expert neurophysiologists, and successfully completed a difficult experimental series in two behaving primates. However, we believe that they have erred in their interpretation that their motor neuron data do not support compartmentalization.

Adade and Das evidently wished to record motor neuron activity under conditions we studied by magnetic resonance imaging (MRI) in humans during vertical fusional vergence (VFV)^3^ and large angle vertical duction.^2^ In the latter paper published in 2016, we explicitly stated predictions for motor neuron behavior that we hoped would be tested by single unit electrophysiological recording of the sort now performed by Adade and Das.^2^ We predicted the following:

1. We predicted that a population of medical rectus (MR) motor neurons, possibly in one subnucleus, will have significant positive correlations with supraduction but abducens neurons will not have such a subpopulation.^2^ The recordings of Adade and Das did support the second part of our prediction, but not the first part. However, we compared the MRI scans of 13 humans during a total prolonged fixations in 46 supraducted with 47 infraducted gaze positions of up to 30 degrees, finding no change at all in overall posterior partial volume (PPV; the measure of contractility) of the MR muscle. Because it required averaging a great deal of MRI data to demonstrate small but statistically significant differences in PPV between the inferior and superior MR compartments during vertical duction, and only relatively weak effects within compartments, we are not surprised that Adade and Das did not find a subpopulation of MR motor neurons in their sample of 26 MR motor neurons of 2 monkeys who made only 15 degrees vertical pursuit movements. Moreover, if such a subpopulation of MR motor neurons were to exist, cell firing features, such as threshold and position sensitivity, might make such neurons difficult to identify as MR motor neurons at all. The failure to identify such neurons in a particular experiment does not prove that such neurons do not exist.
2. We predicted that a population of lateral rectus (LR) motor neurons would show selective sensitivity during vertical vergence.^2^ Figure 3B of Adade and Das shows 2 of 30 LR motor neurons with significant sensitivity to vertical vergence, corresponding to about 7% of all LR motor neurons recorded.^1^ This percentage corresponds closely with the 6% change in PPV of the superior LR compartment we observed by MRI during sustained VFV in humans, as illustrated in figure 7 of our paper.^3^ Adade and Das therefore provide data that seem to us confirmatory of a prediction of the compartmentalization hypothesis.
3. We predicted that “a subpopulation of superior oblique (SO) motor neurons, presumably innervating the (the lateral compartment of the SO muscle) (definitions of acronyms added to quotations for clarity); SOl, will have significant positive correlations with infraduction, but motor neurons innervating the (medial compartment of the SO muscle) SOm will not.” We encourage interested readers to look closely at figure 4C of Adade and Das’ paper. The data in 27 SO motor neurons in figure 4C, pooled from both sides of the brainstem, shows that whereas all of them have about 4 spikes/s/deg sensitivity during vertical pursuit, sensitivity to vertical vergence ranges widely from about 3 to as much as 16 spikes/s/deg, and half of the motor neurons have at least twice the sensitivity to vergence as pursuit. Although this is not exactly what we predicted, it is nonetheless clear that some SO motor neurons with four-fold greater vergence sensitivity are at least relatively selective for vergence, as proposed for compartmentalization. We never suggested that as many as 50% of SO motor neurons would be insensitive to vertical eye position, a clearly exaggerated expectation. In addition, one should bear in mind that the selectivity we have proposed depends on the breadth and location of the SO tendon insertion on the posterior sclera, which is known to vary idiosyncratically among humans, but has not to our knowledge been studied in macaque monkeys. Our third prediction has certainly not been disproven by these data; on the contrary, the prediction seems generally supported.

In their table, Adade and Das made another prediction about infraducting vertical vergence that we did not make: that about half of inferior rectus (IR) motor neurons will have significant vertical sensitivity, and that half will not.^1^ Our MRI study of vertical fusional vergence in 14 humans required only 1.1 degrees of infraduction, and demonstrated 1.3% to 1.4% changes in whole IR PPV, reciprocally in both the infraducting and fellow orbits. In the infraducting eye, the lateral IR compartment exhibited a 1% decrease in PPV, and the medial compartment a 1% increase. We do not understand how our MRI findings would motivate the very strong prediction offered by Adade and Das. But even then, they found that 2 of 46 (4%) IR motor neurons lacked vertical sensitivity during vertical vergence. Perhaps these two neurons are involved in compartmental vergence activity. Given the small PPV changes involved in vertical fusional vergence we found in humans, it is plausible that a subpopulation much smaller than 50% of all IR motor neurons might suffice for this function. Again, the 50% prediction made by Adade and Das seems quite exaggerated in proportion to the MRI behavior we have reported.

We are aware of the daunting challenges involved in single unit electrophysiology in behaving primates, and that this limits the numbers of nonhuman primates that may be studied, and often the types of stimulus conditions that may be used. Nonetheless, these limitations should temper the strength of conclusions drawn from recordings of tens of neurons in only two animals. At least in humans, eye movements, such as vertical fusional vergence, can be highly idiosyncratic. If we had limited our MRI studies to very small numbers of subjects, we could not have demonstrated any significant compartmental effects. In addition, to be considered is the potential effects of differing stimulus conditions. Our MRI studies necessarily involved stable fixations of over 2 minutes each, but during the largest step vergences (1 degree) or gaze eccentricities (30 degrees) that motivated human volunteers could sustain during that time. Adade and Das used gradually changing vergence demand over 60 seconds, or smooth pursuit tracking of smaller (15 degrees) eccentricities for a few seconds.^1^ Thus, Adade and Das did not really replicate the MRI experiments in nonhuman primates, so some differences from our MRI findings would be expected even if differential compartmental muscle behavior occurs under some conditions. Whereas everyone recognizes that it is logically impossible to prove a negative statement, such as Adade and Das’ implication that “EOM compartments are not selectively innervated,” their own demonstration of some motor neuron behavior consistent with the compartmentalization hypothesis would seem to achieve the logical possibility of disproving their negative statement.

The most critical issue involving compartmentalization is the finding that motivated our investigations in the first place: the failure of cyclovertical motor neurons to encode violations of Listing's Law during the vestibulo-ocular reflex,^4^ despite the conformity of eye movements evoked by direct electrical stimulation of the whole abducens nerve.^5^ No other proposed mechanism aside from compartmentalization can explain this otherwise disturbing paradox delinking motor neuron behavior from eye movement behavior. Therefore, we predict that recording of LR motor neuron behavior during a vestibular stimulus, such as ocular counter-rolling, would disclose some units sensitive to head tilt. We hope that Adade and Das or other capable investigators might someday perform this key experiment.

In their Discussion, Adade and Das ignored extensive functional studies from the Demer laboratory showing minimal lateral force transmission among arbitrary groups of bovine EOM muscle^6^ and tendon^7^ fibers during external loading, and EOMs actively contracting ex vivo.^8^ Also ignored was our anatomic evidence suggesting only minimal side-to-side junctions among human extraocular muscle fibers.^9^ These studies provide anatomic and physiological bases for differential compartmental function in human EOMs. On the other hand, the alternative explanations for MRI observations of differential compartmental behavior are entirely speculative, or secondary quotations of speculations. Our PPV analysis of EOM function is taken from the mid to deep orbit, well posterior to the pulleys, so pulleys are unlikely to be the explanation. In addition, whereas the orbit does contain a good deal of pliable fat, there is currently no theory to suggest how fat could change local volumes of particular EOMs only during specific ocular motor behaviors, but not do so in most other cases. Finally, Adade and Das’ data supporting our second prediction above about compartmental LR function in vertical fusional vergence seems to us to be an existence proof that generically refutes all objections to the possibility of differential compartmental function in EOMs.
